# Developing core outcomes sets: methods for identifying and including patient-reported outcomes (PROs)

**DOI:** 10.1186/1745-6215-15-49

**Published:** 2014-02-05

**Authors:** Rhiannon C Macefield, Marc Jacobs, Ida J Korfage, Joanna Nicklin, Robert N Whistance, Sara T Brookes, Mirjam AG Sprangers, Jane M Blazeby

**Affiliations:** 1 School of Social and Community Medicine, University of Bristol, Canynge Hall, 39 Whatley Road, Bristol BS8 2PS, UK; 2 Department of Medical Psychology, Academic Medical Center/University of Amsterdam, Meibergdreef 5, Amsterdam NL 1105 AZ, Netherlands; 3 Department of Public Health, Erasmus MC, P.O. Box 2040, Rotterdam NL 3000 CA, Netherlands; 4 Division of Surgery, Head and Neck, University Hospitals Bristol NHS Foundation Trust, Level 3, Dolphin House, Bristol Royal Infirmary, Marlborough Street, Bristol BS2 8HW, UK

**Keywords:** Core outcome set, Patient reported outcome (PRO), Patient reported outcome measure (PROM), Randomised controlled trial (RCT), Trial methodology, Health domains, Quality of life, Systematic review

## Abstract

**Background:**

Synthesis of patient-reported outcome (PRO) data is hindered by the range of available PRO measures (PROMs) composed of multiple scales and single items with differing terminology and content. The use of core outcome sets, an agreed minimum set of outcomes to be measured and reported in all trials of a specific condition, may improve this issue but methods to select core PRO domains from the many available PROMs are lacking. This study examines existing PROMs and describes methods to identify health domains to inform the development of a core outcome set, illustrated with an example.

**Methods:**

Systematic literature searches identified validated PROMs from studies evaluating radical treatment for oesophageal cancer. PROM scale/single item names were recorded verbatim and the frequency of similar names/scales documented. PROM contents (scale components/single items) were examined for conceptual meaning by an expert clinician and methodologist and categorised into health domains. A patient advocate independently checked this categorisation.

**Results:**

Searches identified 21 generic and disease-specific PROMs containing 116 scales and 32 single items with 94 different verbatim names. Identical names for scales were repeatedly used (for example, ‘physical function’ in six different measures) and others were similar (overlapping face validity) although component items were not always comparable. Based on methodological, clinical and patient expertise, 606 individual items were categorised into 32 health domains.

**Conclusion:**

This study outlines a methodology for identifying candidate PRO domains from existing PROMs to inform a core outcome set to use in clinical trials.

## Background

Outcome selection and reporting in randomised controlled trials (RCTs) is often problematic. Heterogeneity in outcomes measured across studies in the same disease or treatment may hamper effective evidence synthesis. A systematic review of oesophageal studies, for example, found 10 different measures for postoperative mortality which were often undefined [1]. In addition, selective reporting of outcomes puts trials at risk of outcome reporting bias and can mean treatment effects are exaggerated [2]. These issues may be further complicated for patient reported outcomes (PROs). PROs are typically assessed using questionnaires (patient reported outcome measures (PROMs)) and many validated questionnaires are available because PROMs have been developed by different groups and disciplines (for example, clinical *versus* psychological) or for differing purposes (for example, measurement of health in generic populations *versus* disease-specific patient groups). A single PROM can be made up of numerous scales and single items and generic and disease specific PROMs are often combined to assess a range of relevant health domains within an RCT. This means that different (and often ill-defined) outcomes may be reported and the multiplicity of items and scales may also allow selection of statistically significant rather than pre-determined *a priori* PRO endpoints to be reported, increasing the risk of outcome reporting bias. Problems are further accentuated for PROs because terminology of the scales and items across PROMs is not universally agreed meaning data synthesis across studies is difficult when different questionnaires are used, and while there is overlap in the issues that are measured there is also variation because PROMs have been developed by different methods and for different purposes. Potential solutions to these challenges are to develop and use core outcome sets.

Core outcome sets (COSs) are an agreed minimum set of outcome domains to be measured and reported in all trials of a particular treatment or condition [3]. The routine measurement of COSs has the potential to facilitate data synthesis and reduce outcome reporting bias by standardising the outcomes that are measured across studies and this has been emphasised by the COMET (Core Outcome Measures in Effectiveness Trials) initiative which supports the development and application of COSs for pragmatic (effectiveness) trials [4]. Pragmatic trials are designed to assess whether an intervention is effective for routine clinical practice and outcomes, therefore, need to be relevant and important to patients as well as clinicians and other key decision-makers [5]. In many cases these are the outcomes that are assessed with PROMs, particularly if the questionnaire has been developed with patient input [6] but the availability of so many different PROMs, however, means there are problems with selecting which of the measured health domains are ‘core’. The aim of this study, therefore, was to explore and report methods to identify PRO domains from the wealth of available PROMs and to use this approach to inform the development of a COS to use in pragmatic trials in a specific condition. Consensus on which outcomes to include in the final core set, and the methods to achieve this, are the focus of further research.

## Methods

**Figure 1:**
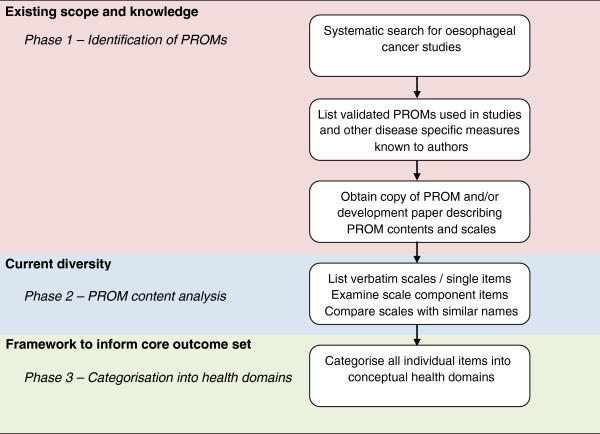
Methods to identify PRO domains to inform a core outcome set.

This study was undertaken within one disease site and treatment - radical treatment for oesophageal cancer, selected because the research team have clinical and PROM expertise in this area and have previously tried to summarise PRO evidence [7-9]. There were three phases of work: (1) a systematic literature review to identify validated PROMs used in oesophageal cancer studies and the scope of these instruments; (2) a detailed content analysis to explore PROM diversity; and (3) categorisation of PROM content into health domains (Figure 1).

### Identification of PROMs used in oesophageal cancer studies

A systematic review was performed to identify and present the scope of existing validated PROMs in order to provide knowledge of the current of state of PRO measurement in this field.

### Search strategy

Electronic searches in MEDLINE, Embase, PsycINFO and CINAHL databases between January 2006 and May 2011 were performed. The search strategy included terms for patient-reported outcomes, oesophageal cancer, surgery and chemotherapy, radiotherapy or combined therapy (see Additional file 1). Searches were limited to studies published in English language. Relevant studies published prior to 2006 were identified from a previous systematic review [8]. Abstracts of identified records were screened for inclusion and full text articles were assessed for eligibility by one of three reviewers (RW, MJ, RCM) with reasons for exclusion documented. No studies were excluded based on a risk of bias assessment or judgement of methodological quality because the purpose of the current study was to identify PROs rather than examine the quality of the data or treatment effect.

### Selection criteria

Included were studies that used at least one validated PROM to evaluate health-related quality of life (HRQL) after radical treatment of oesophageal cancer, including surgical, chemotherapy and/or radiotherapy interventions. Valid PROMs were defined as those that had been tested for psychometric validity and reliability in appropriate patient populations with methodology verified from published papers. No restrictions on study design or sample size were applied. Studies of palliative treatment, comparisons of clinician- or hospital-related factors, and those limited to investigating satisfaction with care or health utilities were excluded.

### Data extraction

Data were extracted using a pre-designed form, piloted before full data extraction with a sample of included studies. Study publication date, design and treatment intervention, the name of the PROM(s), the reported PRO scales and single items, and details of any additional non-validated questions were extracted. These were recorded by one reviewer (RM) and checked by additional members of the study team (MJ, MAGS). The validated PROMs were obtained, including other validated disease-specific PROMs known to authors. Verbatim names for the PRO scales and single items as termed by the PROM developers were extracted and all PROM items (scale components and any single items) were recorded. Data were stored in an electronic database.

### Examination of PROM content

A detailed content analysis of the identified instruments was performed to explore the diversity of PROs in this field. Verbatim names for scales and single items were listed. Scales with identical names and others that were similar (defined as having a least one identical word) were documented, counted and compared for consistency and overlap of the component items.

### Categorisation into health domains

To synthesise the existing content of instruments and provide a framework for future core set development, all PROM items (scale components and any single items) were examined and systematically categorised into conceptual health domains according to the issue they addressed. This was performed by expert methodologists (an oesophageal cancer surgeon and a psychologist) with experience of questionnaire development in health-related quality of life research and cancer (JMB and MAGS) based on their knowledge, familiarity and practiced skill of grouping questionnaire items in this field. Health domains were defined as generic aspects of quality of life affected by health or disease-specific issues and symptoms [10]. Further domains were defined until saturation, that is, all individual PROM items had been mapped onto a domain. Issues addressed in non-validated questions were additionally mapped to domains to verify that the conceptual health domains encompassed all outcomes measured in the included studies. Mapping of items to domains was checked for completeness and consistency by two authors (IK and RCM) and a patient advocate working within oncology research to maximise validity and reliability of the method. Variances were resolved by discussion within the study team and with the senior author (JMB). Data were recorded electronically.

## Results

### Identification of PROMs used in oesophageal cancer studies

A total of 1,351 records were screened for inclusion and 111 full-text articles were assessed for eligibility. Of these, 56 were excluded because they did not meet the criteria for eligibility, including seven studies that used PROMs without sufficient psychometric validation. Some 55 relevant articles reporting 56 studies were identified (Table 1) [11-65]. Almost all studies (*n* = 54, 96%) included data on PROs after surgery, either alone or with neoadjuvant chemo/radiotherapy. Nineteen validated PROMs were used (Table 1) [56,66-83]: nine for gastrointestinal diseases, five cancer-specific instruments and five generic instruments. One oesophageal specific PROM was adapted from a cancer instrument (adapted Rotterdam Symptom Checklist). Three were earlier versions of an updated PROM (EORTC QLQ-C36, QLQ-OES24 and MOS SF20).The most frequently used PROMs were the EORTC QLQ-C30 (*n* = 34, 61%), and the disease-specific modules EORTC QLQ-OES18, or earlier version QLQ-OES24 (*n* = 27, 48%). PROMs were not always used in their entirety, with evidence of selective outcome reporting of scales and single items in 33 (59%), although there was variation across studies in the outcomes that were selected (data not shown). Twenty-one (37%) studies added an additional 74 non-validated items. A further two validated disease specific PROMs; the EORTC QLQ-OG25 [84] and EQOL[85], were sourced from authors’ knowledge, neither of which had been used in a published study since development and validation at the time of the conducted search (May 2011).

**Table 1:** **Oesophageal cancer studies (*n*** **= 56) using validated PROMs (*n*** **= 21)**

		***n***	**(%)**
**Study design**	RCT	4	(7)
	Longitudinal	25	(45)
	Cross-sectional	27	(48)
**Publication year**	<1995	1	(2)
	1995-1999	6	(11)
	2000-2004	12	(21)
	2005-2009	26	(46)
	2010-2011	11	(20)
**Primary outcome**			
Clinical measure (for example, morbidity, mortality)		2	(4)
PRO (for example, HRQL, symptoms)		40	(7)
Undefined/both clinical & PRO		14	(25)
		Studies using PROM (*n*)	PROM scales and single items (*n*)
**PROM**	QLQ-C30	34	15
QLQ-OES18		19	10
SF36		12	8
QLQ-OES24		8	11
GIQLI		3	5
FACT-E		3	5
Adapted RSCL		4	4/5^a^
QLQ-C36		2	18
GERD-HRQL		1	2
DAUGS32		1	7
LAGS		1	3
RSCL		1	3
Adapted RSCL		1	5
PNPC		1	9
WOCS		1	1
MOS SF20		1	6
PAIS		1	7
POMS		1	6
HADS		1	2
EQOL^b^		0	5
QLQ-OG25^b^		0	16

^a^One study used an adapted version with an additional scale [56].

^b^Sourced from authors’ knowledge; PROM yet to be used in a published study at the time of the conducted search.

DAUGS: Dysfunction After Upper Gastrointestinal Surgery**;** EQOL: Esophageal Quality of Life Questionnaire; FACT-E: Functional Assessment of Cancer Therapy - Esophageal; GERD-HRQL: Gastroesophageal Reflux Disease-Health Related Quality of Life; GIQLI: Gastrointestinal Quality of Life Index; HADS: Hospital Anxiety and Depression scale; HRQL: Health-related Quality of Life; LAGS: Life After Gastric Surgery index; MOS: Medical Outcomes Study; OES: Oesophageal; OG: Oesophagogastric; PAIS: Psychosocial Adjustment to Iillness Scale; POMS: Profile of Moods States; PNPC: Problems and Needs in Palliative Care; QLQ: Quality of Life Qquestionnaire; RCT: Randomised controlled trial; RSCL: Rotterdam Symptom Checklist; SF36: Short form 36; WOCS: Worry of Cancer scale.

### Examination of PROM content

There were 116 scales (composed from 574 individual items) and 32 single items in total, with 94 different verbatim scale/item names (Table 2). ‘Pain’ and ‘physical function’ were the most common verbatim name for a scale, used in six different PROMs, but other PROM scale names were also very similar (for example, physical wellbeing, physical problems, physical distress, physical activity, role physical) (Table 3). Some scales with identical names, however, had different component items. For example, ‘physical function’ in one PROM consisted of seven items relating to tiredness/fatigue, feeling unwell, waking up at night, changes in appearance, physical strength, endurance and feeling unfit [72], compared to ‘physical function’ in another PROM consisting of five items that referred to strenuous activity, ability to walk certain distances, time spent in bed or a chair, and need for help with self-care [74]. Similar heterogeneity was found for PROs assessed with single items, for example ‘cough’ in one PROM assessed waking at night because of coughing [67], whereas in another it was an assessment of coughing following eating [69]. While the two items assessed slightly different aspects of coughing they had the same name (‘cough’) and thus reporting would only refer to cough and not the actual issue being assessed within the item.

**Table 2:** Verbatim names of PROM scales and single items

1 Activities of daily living	33 Extended familyrelationships	65 Professional care providers
2 Activity level	34 Fatigue	66 Psychological symptoms
3 Anger-hostility	35 Fatigue-inertia	67 Psychological distress
4 Anxiety	36 Financial difficulties	68 Psychological impact
5 Appetite loss	37 Financial impact	69 Psychological issues
6 Bodily pain	38 Fullness following meals	70 Reflux
7 Body image	39 Functional wellbeing	71 Role emotional
8 Choking	40 General health	72 Role function
9 Cognitive function	41 Global evaluations	73 Role physical
10 Confusion-bewilderment	42 Global QOL	74 Sexual relationships
11 Constipation	43 Global satisfaction	75 Sleep disturbances
12 Cough	44 Global life satisfaction	76 Social activities
13 Deglutition	45 Hair loss	77 Social environment
14 Deglutition disturbances	46 Health perceptions	78 Social function
15 Depression	47 Healthcare orientation	79 Social issues
16 Depression-dejection	48 Heartburn	80 Social/family wellbeing
17 Diarrhoea	49 Indigestion	81 Speech
18 Diarrhoea/constipation	50 Information needs	82 Spiritual issues
19 Difficulty in swallowing	51 Insomnia	83 Stool formation
20 Domestic environment	52 Loss of independence	84 Swallowing problems
21 Dry mouth	53 Loss of weight	85 Symptoms
22 Dumping	54 Medical treatment	86 Symptoms of GERD
23 Dysphagia	55 Mental health	87 Taste
24 Dyspnoea	56 Nausea & vomiting	88 Tension-anxiety
25 Eating	57 Odynophagia	89 Trouble swallowing saliva
26 Eating restrictions	58 Pain	90 Vigour-activity
27 Eating with others	59 Physical symptoms	91 Vitality
28 Emotion	60 Physical activity	92 Vocational environment
29 Emotional function	61 Physical distress	93 Weight
30 Emotional problems (anxiety)	62 Physical function	94 Worry/fear of recurrence
31 Emotional wellbeing	63 Physical problems	
31 Esophageal cancer scale	64 Physical wellbeing	

GERD: Gastroesophageal reflux disease; QOL: Quality of life.

**Table 3:** Identical and similar names for PRO scales used in different PROMs

**Scale name**	**PROMs using identical scale name**	**Scales with similar**^**a **^**names (origin PROM)**
Pain	QLQ C30	Bodily pain (SF 36)
	QLQ C36	
	QLQ OES18	
	QLQ OES24	
	QLQ OG25	
	DAUG 32	
Physical function	MOS SF20	Physical wellbeing (FACT-E)
	QLQ C30	Physical problems (PNPC)
	QLQ C36	Physical distress (RSCL)
	SF 36	Physical activity (DAUG 32)
	EQOL	Role physical (SF 36)
	GIQLI	
Social function	QLQ C30	Social activities (PNPC)
	QLQ C36	Social environment (PAIS)
	SF 36	Social issues (PNPC)
	EQOL	Social/family wellbeing (FACT-E)
	GIQLI	
Activities of daily living	PNPC	
	EQOL	
	RSCL	
Dysphagia	QLQ OES18	
	QLQ OES24	
	QLQ OG25	
Emotional function	QLQ C30	Emotional wellbeing (FACT-E)
	QLQ C36	Emotional problems (anxiety) (QLQ OES24)
	EQOL	Emotion (GIQLI)
		Role emotional (SF 36)
Global QOL	QLQ C30	Global life satisfaction (LAGS)
	QLQ C36	
Nausea & Vomiting	DAUG 32	
	QLQ C30	
	QLQ C36	
Psychological distress	RSCL	Psychological issues (PNPC)
	RSCL(adapted)	Psychological impact (LAGS)
	PAIS	Psychological symptoms (RSCL, adapted)
Reflux	DAUG 32	
	QLQ OG25	
	QLQ OES18	
Role function	MOS SF20	
	QLQ C30	
	QLQ C36	
Symptoms	GIQLI	Physical symptoms (RSCL, adapted)
	LAGS	Symptoms of GERD (GERD-HRQL)
	EQOL	
Activity level	RSCL	Vigour-activity (POMS)
	RSCL (adapted)	
Anxiety	QLQ OG25	Tension-anxiety (POMS)
	HADS	
Cognitive function	QLQ C30	
	QLQ C36	
Eating	QLQ OES18	Eating restrictions (QLQ OG25)
	QLQ OES24	Eating with others (QLQ OG25)
Fatigue	QLQ C30	Fatigue-Inertia (POMS)
	QLQ C36	
Mental Health	MOS SF20	
	SF 36	
Deglutition	QLQ OES24	Deglutition disturbances (DAUG 32)
Depression	HADS	Depression-dejection (POMS)
General health	SF 36	Health perceptions (MOS SF20)

^a^‘similar’ = one or more identical word.

### Categorisation into health domains

All PROM individual items (*n* = 606) were categorised into 32 conceptual generic or symptom specific domains by the study authors (Table 4). Illustrative examples of this categorisation process are provided for some of the generic health domains (Table 5). The most common assessed health domain (concept), that is, the health domain that most PROM items mapped to, was emotional function, assessed in 18 of the 21 PROMs. Other commonly assessed health domains were ‘pain/pain-related swallowing’ (assessed in 14 different PROMs), ‘physical activity/activities of daily life’ (in 13 PROMs) and ‘appetite/eating/taste’ (in 12 PROMs). Uncommon domains were ‘spiritual issues’ (assessed in one PROM) and ‘dizziness/dumping’ (assessed in two PROMs). Non-validated questions predominantly focused on eating and therefore were mapped onto the ‘appetite/eating/taste’ domain. A patient advocate checked the categorisation of items into health domains and there were no difference of opinion.

**Table 4:** Categorised PRO health domains showing number of items in existing PROMs assessing each domain

	**Specific measures for gastrointestinal disease**											**Cancer specific measures**									**Generic measures**	
PRO domain	EQOL	FACT-E	GERD-HRQL	GIQLI	DAUGS 32	LAGS	QLQ-OG25	QLQ-OES18	QLQ-OES24	Adapted RSCL^a^	Adapted RSCL^b^	RSCL	PNPC	QLQ-C30	QLQ-C36	WOCS	MOS Sf-20	PAIS	POMS	SF 36	HADS	Frequency assessed in PROMs (total number of items)
*Disease specific*																						
Pain/pain-related swallowing		3	1	1	2	1	3	3	3		5	6	1	2	1		1			2		15 (35)
Appetite/eating/taste	4	3		4	5	1	6	4	4		3	1	1	1	1							13 (38)
Fatigue		1		1	2	1					1	2	1	3	3				15	4		11 (34)
Dysphagia/swallowing saliva	1	4	1	1	2	1	4	4	4		2		1									11 (25)
Regurgitation/vomiting	1			1	5	1	1	1	1		2		1	1	1							11 (16)
Reflux/heartburn	1		6	1	2	1	1	1	1		1	1										10 (16)
Nausea		1		1	1	1					1	1	1	1	1							9 (9)
Belching/bloating/gas	1		1	4	3	1			1		1											7 (12)
Diarrhoea/frequent bowel movements				5	2	1					1		1	1	1							7 (12)
Cough		1				1	1	1	1		1		1									7 (7)
Choking		1			1	1	1	1	1		1											7 (7)
Dry mouth		1					1	1	1		1	1										6 (6)
Breathing		1									1	1	1	1	1							6 (6)
Weight	1	1			1	1	1		1													6 (6)
Communication/speech difficulties		2					1	1	1		1											5 (6)
Eating - social impact	1	1					1	1	1													5 (5)
Sexual function				1								1	1					6				4 (9)
Constipation				1									1	1	1							4 (4)
Hair loss							1		1			1	1									4 (4)
Body image				1			1						1					1				4 (4)
Dizziness/dumping					5	2							1									3 (8)
*Generic*																						
Emotional function	2	8		6		3	2		3	7	5	7	19	4	8	4	5	6	43	5	14	18 (151)
Role physical/ADLs	2	2	1	2						8	8	8	9	3	4		3	4		5		13 (59)
Physical function		2		3	1					25		3	4	4	6		5	1		9		11 (63)
Social function	1	7		1									19	2	2		1	17		2		9 (52)
Generic health		1		1										1	1		5	2		6		7 (17)
Sleep		1		1		1						1	1	1	1							7 (7)
Global QOL		1								1	4		1	1	2							6 (10)
Cognition												1	3	2	1				7			5 (14)
Role emotional		2											4					2		3		4 (11)
Financial issues													5	1	1			1				4 (8)
Spiritual issues													2									1 (2)

^a^Adapted RSCL - de Boer study.

^b^Adapted RCSL - van Knippenberg study

ADLs: Activities of daily life.

**Table 5:** Selected categorised health domains and example PROM items mapped to these domains

**PRO domain**	**Example items (origin PROM)**
Emotional function	Did you feel tense? (QLQ-C30)
	I feel sad (FACT-E)
	How often in the past two weeks have you felt fearful of cancer recurrence? (EQOL)
Role physical/ADLs	Has your illness interfered with your ability to do your job? (PAIS)
	If you take medicine, does this affect your daily life? (GERD-HRQL)
	Does your health now limit you in bathing or dressing yourself? (SF 36)
Physical function	Because of your illness, how much physical strength have you lost? (GIQLI)
	Did you have any trouble taking a long walk? (QLQ-C30)
	I am forced to spend time in bed (FACT-E)
Social function	How limited have you been in the past two weeks visiting friends or relatives? (EQOL)
	To what extent have your personal relations with people close to you worsened because of your illness? (GIQLI)
	Are you still as interested in your leisure time activities and hobbies as you were prior to your illness? (PAIS)
Generic health	How often during the past two weeks have you felt unwell (GIQLI)
	How would you rate your overall health during the past week? (QLQ-C30)
	I seem to get sick a little easier than other people (SF 36)

ADLs: Activities of daily life.

## Discussion

This study comprehensively analysed PROs from studies in radical treatment for oesophageal cancer. Some 116 scales and 32 single items were identified from 21 validated PROMs. As many as 94 different verbatim names were used to describe PRO scales and single items and although many names were similar, content examination revealed component questions did not always address comparable issues. In-depth examination and categorisation of PROM contents concluded that together they addressed 32 different health domains demonstrating the vast overlap between PROMs.

Our findings show how evidence synthesis of oesophageal cancer PROs may be hampered because of the range of PROMs used in trials and the multiple scales and single items within them, often with inconsistent and non-transparent terminology. Core outcome sets aim to reduce this problem by identifying and prioritising the important health domains to be measured in all studies. The development of core outcome sets in other clinical areas has been undertaken using a range of methods, in particular the approach to including PROs [86-89]. In rheumatoid arthritis, for example, the initial American College of Rheumatology (ACR) core set was developed by a committee of experts (16 professionals in rheumatoid arthritis trials, health services research and biostatistics) who reviewed the literature on the validity of trial outcomes (for example, sensitivity to change or how well it predicted/correlated with a definite clinical change) and used a nominal group process to recommend and reach consensus on a list of core outcomes. The list was presented and finalised at a specialist international conference (OMERACT: Outcome Measures for Rheumatology in Clinical Trials) and contained both clinical and PROs, although patients were not involved in the consensus process. Outcomes were specific (for example, number of swollen joints) or more general domains (for example, functional status), with recommendations on how to measure the outcomes decided later [86]. Subsequent OMERACT conference discussions and workshops deliberately involved patients and led to the addition of fatigue in the ACR core set [87,90], and continued work using interviews with patients, identified further important PROs [5]. This led to the development of a ‘patient core set’ of disease-specific and global outcome domains solely derived from patient opinion to complement the professional ACR core set [88]. Our current study methodology ensures that the patient perspective and relevant PROs inform the development of a core set of outcome domains from an early stage, because it examines the content of validated PROMs which are developed with significant patient involvement. The identified PRO domains will be prioritised using a Delphi method to reach consensus on the important to include in the core outcome set, alongside clinical outcomes [1], and is the focus of future work. Patients, surgeons and clinical nurse specialists will be surveyed to ensure the opinions of all key stakeholders are sought, a recommended approach by the COMET (Core Outcome Measures in Effectiveness Trials) initiative [4].

This study included a detailed systematic search to identify PROs measured in oesophageal cancer studies and used rigourous methodology to identify health domains, however, it does have weaknesses. The categorisation of question items into health domains was performed by two experts and independently checked by other members of the research team, including a patient advocate, but it is possible that others may have categorised items differently. Inter-rater reliability statistics could have been recorded to describe agreement between the experts when categorising items. Future work therefore is needed to standardised and validate this method. In addition, presentation of the methodology to a greater number of patients or patient representatives could strengthen the robustness and reliability of the categorisation process.

## Conclusion

In summary, this study demonstrates there is diversity in the PROMs selected to evaluate radical treatment for oesophageal cancer. Within and between PROMs there is a lack of clarity between named scales and items and the underlying health domains being assessed meaning data synthesis is limited. A methodology for identifying important PRO health domains is proposed which can be used to inform the development of a core set of health domains. Following this it will be necessary to determine accurate and efficient ways to measure these core domains, drawing on items banks developed by initiatives such as PROMIS (Patient Reported Outcomes Measurement Information System) and COSMIN (Consensus-based Standards for the selection of health status Measurement Instruments) [91,92].

## Abbreviations

COS: Core outcome set; HRQL: Health-related quality of life; PRO: Patient-reported outcome; PROM: Patient-reported outcome measure; RCT: Randomised controlled trial.

## Competing interests

The authors declare they have no competing interests.

## Authors’ contributions

RM conducted and coordinated the review and drafted the manuscript. MJ was involved in the systematic databases searches, data extraction and contributed to the draft manuscript. IK and JN contributed to the systematic search for studies. RW helped to screen abstracts for the review. ST contributed to the design of the study. MS checked the data extraction, grouped data into PRO domains and contributed to the draft manuscript. JB conceived the idea for the review, was involved in the design of the study and data extraction, grouped data into PRO domains, contributed to the draft manuscript and supervised the project. All authors read and approved the final manuscript.

## Supplementary Material

Additional file 1 Search strategy as applied to MEDLINE (OVID). Click here for file
