# Supplementary material for: Developing core outcomes sets: methods for identifying and including patient-reported outcomes (PROs)
Source: Trials. 2014 Feb 5;15:49. doi: 10.1186/1745-6215-15-49 (PMC3916696; doi:10.1186/1745-6215-15-49)
Supplement: Additional file 1 — Search strategy as applied to MEDLINE (OVID). [file 1745-6215-15-49-S1.doc]

Additional file 1 Search strategy as applied to MEDLINE (OVID)

| Oesophageal cancer |
| --- |
| 1. explode Esophageal neoplasms/ |
| 2. (oesophag$ adj5 (cancer$ or tumo?r$ or neoplasm$ or carcinoma$ or malignan$)).tw. |
| 3. (esophag$ adj5 (cancer$ or tumo?r$ or neoplasm$ or carcinoma$ or malignan$)).tw. |
| 4. oesophag$ adj5 adenocarcin$).tw. |
| 5. (esophag$ adj5 adenocarcin$).tw. |
| 6. (oesophag$ adj5 squamous$).tw. |
| 7. (esophag$ adj5 squamous$).tw. |
| 8. Barretts adj5 oesophag$.tw |
| 9. Barretts adj5 esophag$.tw |
| 10. or/1-9 |
| Surgery |
| 11. Esophagectomy/ |
| 12. esophagectomy.tw |
| 13. esophagectomy$5 surgery.tw |
| 14. esophagectom$5.tw |
| 15. Esophagectom$ adj5 surgery.tw |
| 16. esophageal.tw |
| 17. oesophagectomy.tw |
| 18. oesophagectomy$5 surgery.tw |
| 19. oesophagectom$5.tw |
| 20. oesophagectom$ adj5 surgery.tw |
| 21. oeosophageal.tw |
| 22. oesophageal adj5 surgery.tw |
| 23. esophageal adj5 surgery.tw. |
| 24. or/11-23 |
| Chemotherapy, radiotherapy or combined therapy |
| 25. explode combined modality therapy/ |
| 26. explode drug therapy combination/ |
| 27. explode chemotherapy adjuvant/ |
| 28. explode radiotherapy adjuvant/ |
| 29. chemotherapy.tw. |
| 30. radiotherapy.tw. |
| 31. explode Radiotherapy/ |
| 32. or/25-31 |
| Patient reported outcomes |
| 33. Quality of Life/ |
| 34. quality of life.tw. |
| 35. qol.tw. |
| 36. hrql.tw. |
| 37. hrqol.tw. |
| 38. Outcome Assessment (Health Care)/ |
| 39. explode Outcome Assessment (Healthcare)/ |
| 40. patient reported outcome.tw. |
| 41. Health Status/ |
| 42. health status.tw. |
| 43. lifestyle.tw. |
| 44. questionnaire.tw. |
| 45. Questionnaires/ |
| 46. or/33-45 |
| 47. 10 and 24 and 46 |
| 48. 10 and 32 and 46 |
| 49. 47 or 48 |
| 50. Limit 49 to (abstracts, english language and humans and yr=’2006—Current’) |
